# Computational deconvolution of DNA methylation data from mixed DNA samples

**DOI:** 10.1093/bib/bbae234

**Published:** 2024-05-18

**Authors:** Maísa R Ferro dos Santos, Edoardo Giuili, Andries De Koker, Celine Everaert, Katleen De Preter

**Affiliations:** VIB-UGent Center for Medical Biotechnology (CMB), Technologiepark-Zwijnaarde 75, 9052 Zwijnaarde, Belgium; Cancer Research Institute Ghent (CRIG), 9000 Ghent, Belgium; VIB-UGent Center for Medical Biotechnology (CMB), Technologiepark-Zwijnaarde 75, 9052 Zwijnaarde, Belgium; Cancer Research Institute Ghent (CRIG), 9000 Ghent, Belgium; VIB-UGent Center for Medical Biotechnology (CMB), Technologiepark-Zwijnaarde 75, 9052 Zwijnaarde, Belgium; Cancer Research Institute Ghent (CRIG), 9000 Ghent, Belgium; VIB-UGent Center for Medical Biotechnology (CMB), Technologiepark-Zwijnaarde 75, 9052 Zwijnaarde, Belgium; Cancer Research Institute Ghent (CRIG), 9000 Ghent, Belgium; VIB-UGent Center for Medical Biotechnology (CMB), Technologiepark-Zwijnaarde 75, 9052 Zwijnaarde, Belgium; Cancer Research Institute Ghent (CRIG), 9000 Ghent, Belgium

**Keywords:** DNA methylation profiling, computational deconvolution, tool comparison

## Abstract

In this review, we provide a comprehensive overview of the different computational tools that have been published for the deconvolution of bulk DNA methylation (DNAm) data. Here, deconvolution refers to the estimation of cell-type proportions that constitute a mixed sample. The paper reviews and compares 25 deconvolution methods (supervised, unsupervised or hybrid) developed between 2012 and 2023 and compares the strengths and limitations of each approach. Moreover, in this study, we describe the impact of the platform used for the generation of methylation data (including microarrays and sequencing), the applied data pre-processing steps and the used reference dataset on the deconvolution performance. Next to reference-based methods, we also examine methods that require only partial reference datasets or require no reference set at all. In this review, we provide guidelines for the use of specific methods dependent on the DNA methylation data type and data availability.

## INTRODUCTION

DNA methylation (DNAm) involves modifying DNA epigenetically by covalently adding a methyl group to the 5′ position of the pyrimidine ring of cytosine residues within CpG dinucleotides. This modification is commonly found in CpG islands, situated predominantly in and around promoter regions, and the frequency or proximity of these modifications can impact the transcriptional process. Methylation of these promotor regions affects the interaction of the transcriptional machinery with the DNA and generally results in transcriptional silencing of genes [1]. Due to its regulatory function, DNAm plays a key role in normal cell development and differentiation [2, 3]. Distinct cell types can be recognized by their unique DNAm pattern [4–6], reflecting the cell-type-specific transcriptional programme influenced by this epigenetic layer.

Modifications in these DNAm patterns may lead to many diseases, including cancer [4, 7]. These patterns can serve as diagnostic markers for various diseases, demonstrated by several studies [8–10], where DNAm was used to identify disease-specific epigenetic signatures, called epi-signatures. Classification of brain tumours based on bulk DNAm profiling is another successful clinical application of this concept. Certainly, modern diagnostics can accurately identify specific types of brain tumours by leveraging their unique DNAm profile [11]. However, these diagnostic approaches primarily utilize bulk DNAm profiling methods that, while clinically useful in capturing the dominant signal predominantly emanating from the overgrown cell type, cannot investigate complex samples at a cellular resolution [12, 13]. Several works show that distinguishing methylation profiles of cell types (e.g. to estimate tumour percentages or study the immune context) in mixed cell populations is challenging. Indeed, to properly identify the cell-type composition of a sample single-cell methylation profiling tools are required, but these are currently very costly, therefore difficult to scale, and often generate noisy and sparse data [14]. For this reason, several computational approaches have been developed during the past years to infer the abundance of different cell types in heterogeneous samples. This task is known as computational deconvolution of DNAm data from mixed samples. The first computational deconvolution methods were developed for RNA expression data (reviewed in Avila-Cobos *et al*. [15]). In 2012, the first deconvolution algorithm for DNA-methylation data was published [16], after which several other methods were reported and all these are reviewed in this manuscript.

In this review, we explain the deconvolution problem, define the criteria for the deconvolution methods that will be discussed here, describe the features that affect the performance, give a detailed description and evaluation of each method, propose a classification of the methods according to their reference dataset dependence and summarize some guidelines for the selection of a method dependent on the research question to be answered.

### The deconvolution problem

Computational deconvolution infers relative cell-type proportions present in a mixed sample, such as tissue or blood. In mathematical terms, this problem can be formulated as the following linear equation of matrices:


\begin{align*} T=(CP)+\varepsilon \end{align*}

\begin{align*} \left\lceil \begin{array}{ccc}{T}_{11}& \cdots & {T}_{1n}\\{}\vdots & \ddots & \vdots \\{}{T}_{g1}& \cdots & {T}_{gn}\end{array}\right\rceil =&\left(\left\lceil \begin{array}{ccc}{C}_{11}& \cdots & {C}_{1m}\\{}\vdots & \ddots & \vdots \\{}{C}_{g1}& \cdots & {C}_{gm}\end{array}\right\rceil\right. \\&\times \left.\left\lceil \begin{array}{ccc}{P}_{11}& \cdots & {P}_{1n}\\{}\vdots & \ddots & \vdots \\{}{P}_{m1}& \cdots & {P}_{mn}\end{array}\right\rceil \right)+\left\{{\varepsilon}_1,\dots, {\varepsilon}_n\right\} \end{align*}

Where $T$ is the methylation matrix ($g\times n)$ containing methylation data of g regions in n mixed samples; $C$ is a matrix ($g\times m)$ that comprises the methylation data of the m different cell entities for g regions (also called the reference dataset); $P$ is a matrix$\left(m\times n\right)$ containing the proportions of *m* cell entities in n mixed sample; and $\varepsilon$ is a non-negative vector of size $n$ representing the signal’s noise in $T$. When applying reference-based methods, both $T$ and $C$ are known variables and the cell proportions in P must be estimated. In reference-free methods, only T is known, which makes it more complex to solve.

### Selection of methods in this review

In the past years, a few dedicated reviews/benchmarking studies on computational deconvolution of DNA-methylation data were published: Teschendorff [17] (10 methods), Titus [18] (10 methods), Scherer [19] (14 methods), Jeong [20] (8 methods), Song [21] (6 methods) and Sharma [22] (11 methods). Combined, these studies summarize a total of 28 tools, methods or method variations for deconvolution of DNAm data. However, it is important to note that 13 out of these 28 methods do not estimate cell-type abundance. Instead, they focus on tasks such as correcting cell-type composition heterogeneity by eliminating confounding factors or noise, identifying significant methylation sites (SVA [23], ISVA [24], RUV-2 [25], EWASher [26], ReFACTor [27], ICA [28], TCA [29]) or are limited to feature selection as a preliminary step before actual deconvolution (MethylCIBERSORT [30], CONFINE [31], csmFInder/coMethy [32], TOAST [33] and ClubCPG [34]). Additionally, one of the methods was specifically designed for gene expression data and not applicable to DNA methylation analysis (CDSeq [35]), bringing to a total of 14 tools that fall outside our definition of DNA methylation deconvolution.

**Figure 1:**
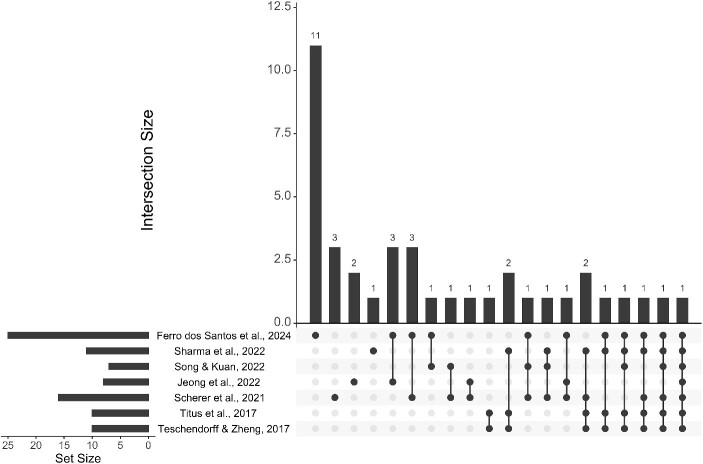
UpSet plot displaying the number of tools/methods covered in the different reviews/benchmarking studies. This review involves 11 methods that have not been reviewed before. The first described method (the Houseman’s reference-based approach [13]) is included in all the reviews.

Through an extensive literature review, we identified 11 additional methods that allow deconvolution of DNAm data, and we evaluated them together with the 14 previously reviewed methods (# Figure 1 #). In addition, we shortly discuss four methods that, according to our definition, are not strict deconvolution methods but they allow us to identify the main contributing cell type in a mixture, being designated as classification tools instead. All methods reviewed or indicated in this manuscript are summarized in Supplementary File 1.

### Parameters affecting deconvolution performance

Apart from the method/algorithm selection, several parameters might influence the deconvolution results’ accuracy and performance.

Some assumptions made in the algorithms might influence the performance, for example, the number of entities present, the linearity of relationships between methylation and these entities and the assurance that the sum of the cellular fractions equals 1. Different models may exhibit varied performance across different biological conditions, depending on the input data chosen or the user-defined tool parameters, and, therefore, their selection significantly influences the results.

Although the effect, as far as we could ascertain, has not yet been thoroughly studied, we summarize here some important parameters that need to be considered for the experimental design of a DNAm deconvolution study.

1) Platform and technology utilized for DNAm data generation: The choice of technology, whether array-based or sequencing-based, introduces inherent variations in DNAm measurements. Array technologies may exhibit platform-specific biases and limitations, such as probe design constraints that capture methylation events only in specific genomic regions [36]. In contrast, most sequencing-based methods provide a more comprehensive view of DNAm patterns but may also entail their own set of biases and limitations, including differences in read depth and coverage across the genome. These platform-specific differences underscore the importance of considering the nuances and potential biases introduced by the chosen technology when analysing and interpreting DNAm data.
2) Data quality and pre-processing steps: Factors such as noise, missing data, batch effects or technical artifacts can significantly impact data quality. In this regard, data pre-processing, which includes normalization, mapping, filtering and feature selection, becomes essential to mitigate these issues. However, it’s important to note that different data preprocessing methods will result in varying data transformations, thereby influencing the accuracy of the final results. [36, 37].
3) Reference profile quality and completeness: Also, the quality and purity of the used reference dataset (matrix C in equation) will affect the accuracy of the results [20]. The completeness of the reference dataset is another critical factor in deconvolution analyses. Typically, having a comprehensive reference dataset including all cell types contributing to the mixed sample will improve the deconvolution outcomes of these samples. Thus, the completeness of this dataset is crucial to ensure accurate capturing of the complex cellular composition in mixed samples
4) Sample size and resolution: The implementation in large sample cohorts generally provides more robust and accurate deconvolution results, increasing the statistical power of the analysis. There is also the case where partial-reference tools were designed to fit multiple samples simultaneously since the model will learn the custom unknowns from the previous samples [38]. The accuracy of DNA methylation data in deconvolution analyses can also be linked to its resolution, determined by the quantity of individual reads and the number of CpG sites measured. A higher resolution, marked by an increased number of reads and CpG sites, can in some cases significantly enhance the precision of deconvolution results. This is attributed to the detailed and comprehensive depiction of DNA methylation patterns, enabling a more nuanced evaluation of cell-type proportions in mixed samples. Conversely, lower-resolution data may lack the necessary granularity to discern subtle methylation differences, potentially leading to less accurate estimations of cell-type contributions. Optimizing methylation data resolution by maximizing reads and CpG sites has the potential to improve the accuracy and reliability of deconvolution analyses, depending on the methods applied [39].
5) Similarity of the contributing cell types: When certain cell types have a more alike methylation profile, such as in the case of certain tumour subtypes, deconvolution might become more challenging for these cell types [40].
6) Cell-type composition: Extreme or unbalanced cell-type compositions within a sample might pose challenges for deconvolution algorithms, especially when certain cell types are scarce or overly abundant [41].
7) Inter-individual variability: Methylation patterns within DNA are susceptible to influences stemming from individual differences, encompassing factors such as age, sex, smoking status, overall health and dietary habits, among others. Given the diverse array of elements that can impact methylation, it becomes crucial to consider the selection of reference datasets that are well-matched in terms of age, sex and other pertinent characteristics when conducting deconvolution on a specific set of mixed samples. By ensuring that the reference dataset aligns closely with the demographic and physiological attributes of the samples under investigation, the deconvolution process becomes more adept at accurately capturing the underlying cellular composition. This attention to demographic and biological matching enhances the applicability and reliability of deconvolution outcomes, accounting for potential confounding factors and refining the precision of cell-type proportion estimations in complex sample mixtures [42–45].

### Preparing input data for deconvolution

In this paragraph, we discuss what platforms can be selected to generate data. We also describe the pre-processing steps and the different data formats that can be used as input for the deconvolution pipelines.

### DNAm platforms

Before the introduction of sequencing-based DNAm profiling methods, methylation arrays were mostly used in studies including EWAS (epigenome-wide association studies) [13]. In array-based approaches, probes are used to capture DNA fragments to detect the methylation status of specific CpG sites or regions across the genome [46], providing an intensity for both methylated and unmethylated CpG sites covered by the probes. Different microarray platforms, each with their specific probe sets, are available [47], including Infinium’s HumanMethylation450 BeadChip and MethylationEPIC BeadChip from Illumina [12]. These technologies are still widely used to date due to their high reproducibility, ease of analysis and considerable sensitivity and specificity at a lower cost [36, 48]. Despite their ability to detect significant methylation changes, these technologies exhibit a lower resolution due to the limited number of probes compared to the, even genome-wide, resolution achieved by most sequencing-based methods.

Over the past few years, a multitude of sequencing-based technologies have emerged [13], each employing unique experimental approaches to discriminate or capture methylated versus unmethylated CpGs: (oxidative) bisulphite conversion (whole genome bisulphite sequencing: WGBS), enzyme-conversion (enzymatic methylation sequencing: EM-Seq), methylation-specific restriction (Methyl-Seq/MRE-Seq) and affinity enrichment (antibody-based approaches) and capture-based methods [13, 49] (like MethylCap-seq). Upon bisulphite or enzymatic conversion of unmethylated CpGs, sequencing of the converted genome can be performed genome-wide such as in WGBS or whole genome EM-seq. However, these methods are costly and therefore approaches exist to capture specific regions using hybridization-based methods [50] or to enrich for CpG-rich regions using MspI digestion (in methods like reduced representation bisulphite sequencing, RRBS) [51].

Another recently developed enzyme-based approach is the six-letter sequencing that allows sequencing of genetic and epigenetic bases (5mC and 5hmC) of DNA samples in a single workflow [52].

Also recently, nanopore sequencing entered the field. This method relies on a difference in the electric current flow when a specific genetic/epigenetic base passes the nanopore. As such the pores can detect several epigenetic base modifications including DNA-methylation [53].

A breakdown of the general advantages and disadvantages of the most adopted DNA methylation profiling methods is provided in Supplementary File 2.

In a deconvolution study the reference dataset and the test dataset are ideally generated on the same methylation profiling platform. However, deconvolution is also possible in mixed-platform experiments such as in the study of Moss *et al.* [54] where data from Infinium 450K and EPIC array platforms are combined or in the study of Van Paemel *et al.* [4] where a combination of cfRRBS, WGBS, Infinium and EPIC array data was utilized.

### Data pre-processing

The different pre-processing steps used to analyse the raw methylation array or sequencing data also impacts the deconvolution performance. In the case of methylation arrays, fluorescent intensity measurements are converted to relative abundances of methylated and unmethylated cytosines [37, 55] in several steps: quality control, data filtering (e.g. elimination of non-autosomal chromosomes and low signal probes), normalization, mapping or matching of probes to genomic coordinates (using annotation packages) [56, 57]. Another pre-processing step that can be conducted prior to the deconvolution is an optional probe-wise differentially methylated regions (DMRs) analysis.

Pre-processing of sequencing data (e.g. bisulphite sequencing data) involves adaptor trimming, quality control, alignment to a reference genome, methylation calling and individual CpG or CpG cluster filtering [4, 58, 59]. CpG filtering might imply removal of CpGs linked to non-autosomal chromosomes (e.g. the X and Y chromosomes) and of known sex-associated CpGs on the autosomal chromosomes [60, 61], to minimize sex-related biases, or removal of CpGs located on known single-nucleotide polymorphism (SNP) positions [62] to reduce the effects of genetic variability between the samples.

Instead of using the whole methylation profile for deconvolution, most tools benefit from the selection of the most relevant and cell type–specific markers (tissue informative markers (TIMs) [38]). This process helps to differentiate between similar cell types and reduces the computational intensity of the deconvolution [20, 21]. These markers can be identified using differential methylation analysis (either at a probe location, CpG or CpG cluster level) using tools such as DMRfinder [63], DSS [64] or MethylCIBERSORT [30], to name a few. Other approaches to identify tissue-specific methylation markers are the selection of the most variable regions (such as the specific hyper- and/or hypomethylated regions) [54, 65], the use of (moderated) *t*-tests [3, 60, 66–68] or F statistics (ANOVA) [16].

### Input data types for deconvolution

Most deconvolution tools/methods described in this review use beta values as input data. DNAm beta values are continuous variables between 0 and 1, representing the percentage of methylation or methylated cytosines measured in a certain region (arrays) or at a certain CpG site (sequencing), respectively.

Some weighted deconvolution methods, such as CelFiE [38] and ARIC [69], require the absolute count of methylated and unmethylated reads for each CpG as input data, since these methods attribute more weight to highly covered CpGs in the sequencing data.

The *PRISM* [65] tool requires mapped data as input in the form of BAM files.

More recently, some methylation deconvolution algorithms have started using alpha values instead of beta values [70–72]. These values can only be obtained from methylation sequencing data and correspond to a read-based measure of methylation instead of CpG-based. Alpha values are the number of methylated CpG sites divided by the total number of CpG sites on a read and thus range from 0 to 1. Similarly, the authors of *UXM* [65] developed a fragment-level deconvolution algorithm which stores the read-level methylation information (included in the .bam files) into a specific DNAm file format (.pat file). Each fragment (read) is annotated as U (mostly unmethylated), M (mostly methylated) or X (mixed) depending on the number of CpGs methylated or unmethylated. The deconvolution is then performed based on the proportion of U fragments.

The input data types for different deconvolution methods are summarized in Table 1.

**Table 1:** Types of data used as input for the deconvolution analysis. In the reviewed tools, 5 types of input data were distinguished, described, and associated with their respective users.

Input data type	Tools
Absolute count of methylated (M) and unmethylated (U) cytosines	CelFiE, ARIC
Mapped data (BAM files)	PRISM
Alpha values	CelFEER, cfSort
Read-level methylation information (PAT files)	UXM
Beta values	All the other tools included in the review

### Deconvolution tools

**Figure 2:**
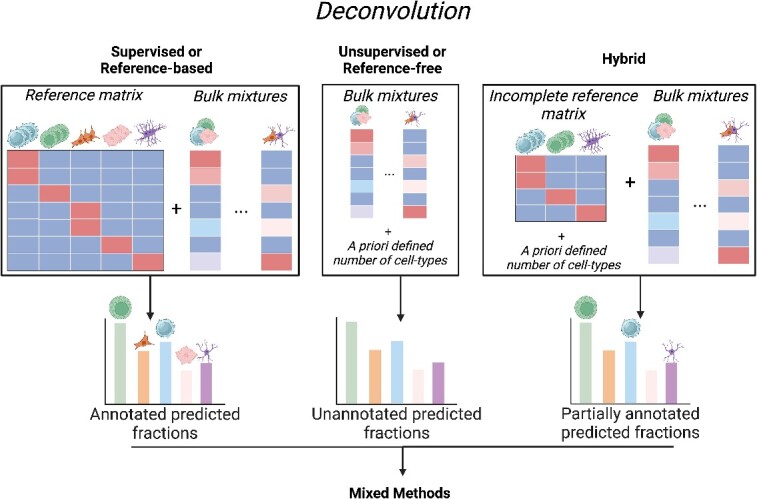
Deconvolution tools can be subdivided into four categories: Supervised or Reference-based, Unsupervised or Reference-free, Hybrid and Mixed. For the last three categories, the a priori defined number of cell types will determine the total number of entities presented in the results. Mixed methods, depending on the settings, have the capability to perform deconvolution under various conditions [unsupervised or (semi-)supervised] (Created with BioRender.com).

Deconvolution tools and algorithms are typically classified as supervised (reference-based) or unsupervised (reference-free) methods depending on their need for reference methylation profiles of the contributing cell types. Some of the more recent methods can be classified as hybrid: these methods use incomplete reference sets that do not encompass all the expected cell types within the mixture, or blend aspects of unsupervised approaches, preserving a certain level of adaptability, while also integrating prior knowledge, such as differentially methylated regions/positions for the diverse entities or cell types anticipated within mixed samples (Figure 2).

### Supervised or reference-based methods

Reference-based techniques are widely used for DNAm deconvolution. These approaches utilize a collection of methylation profiles from a range of healthy or diseased cells or tissue entities that contribute to the mixture. The reference set is used to estimate the composition of the mixed samples, providing information about the proportions of distinct cell types.

Different methodological approaches exist to accurately estimate the fractions of the different cell types represented in the reference set. Based on the mathematical computational method behind the approach, we can distinguish:

1) The original Houseman’s approach [16] is a linear Constrained Projection-based (CP) algorithm and the first deconvolution method developed for methylation data. This technique estimates proportions by minimizing differences between observed mixed-sample methylation data and the reference matrix’s projection onto the cell-type proportion matrix while maintaining specific constraints. The CP method relies thus on characterized DNAm profiles from distinct cell types. Through optimization, it solves the equations that aligns methylation data from a mixed sample with these reference profiles, by ensuring that the resulting cell-type proportions are non-negative and possibly summing to 100% (dependent on the used equality constraint). As such, the proportions of the samples are predicted.
2) Least Square (LS) Regression models seek to establish an optimal linear relationship between predictors (in this case, the methylation profiles of the reference) and a response variable (here the profiles of the mixed samples) by minimizing the squared differences between the observed and predicted values. Solving this alignment computes coefficients indicating the contributions of individual cell types to the mixed profile. Consequently, LS Regression facilitates inferring cell-type proportions from complex mixtures based on methylation patterns, depending on the quality of reference profiles and the assumption of linear associations between methylation levels and cell-type proportions. Methods such as MethAtlas [54], UXM [65] and MethylResolver [60] are based on these models. The first two methods employ Non-Negative Least Square (NNLS) Regression, and the third method makes use of Least Trimmed Squares (LTS) Regression.
3) Robust Partial Correlation-based (RPC) models are used in one of the algorithms implemented in the EpiDISH package [68] (RPC—Least Squares) and in the *EPISCORE* method [73] (weighted RPC), where the latest uses a combination of scRNA-seq and DNAm data as input for the deconvolution. These RPC models are, as the name mentions, robust techniques that assess correlations between variables (methylation data) while considering other factors (external influences, conditions or characteristics affecting the observed correlations, such as subject sex and age) in the model and addressing potential outliers or non-standard data behaviour. RPC methods, unlike traditional partial correlation approaches, prioritize accuracy, particularly in the presence of noisy or contaminated datasets. Using robust statistical measures, RPC methods enhance the reliability of assessing relationships between variables, especially in scenarios involving non-standard data behaviours or outliers that could distort correlation analyses. Applied to DNAm data for deconvolution, RPC methods estimate cell-type proportions within mixed samples by leveraging partial correlation analysis between methylation patterns at different CpG sites while accounting for outliers and noise.
4) Support Vector Regression (SVR) models, when applied to DNA methylation data for deconvolution purposes, are machine learning algorithms that aim to predict cell-type proportions within mixed samples based on DNA methylation patterns. SVR operates by finding an optimal hyperplane that best represents the relationship between the methylation data and cell-type proportions. This method uses support vectors to define the hyperplane, which maximizes the margin between different data points while minimizing prediction errors. SVR models learn from the methylation data, mapping it to the known cell-type proportions in the training dataset, and then predict the proportions of cell types in new or unseen samples. By leveraging SVR in DNA methylation analysis, these models attempt to accurately estimate cell-type contributions within mixed samples, offering predictive capabilities that aid in deconvolution and understanding cellular compositions in complex biological systems. CIBERSORT [74] (ν-SVR), CIBERSORTx [75] (SVR with elastic net) and ARIC [69] (weighted υ-SVR) are examples.
5) Deep neural network (DNN) models, like in cfSort [71], are sophisticated machine learning algorithms characterized by multi-layered neural network architectures that learn complex representations from data. Comprising interconnected nodes arranged in input (methylation profiles), hidden and output layers (proportions) with weighted connections, DNNs adjust these weights via backpropagation during training (with sets of pure samples of known origin) to minimize prediction errors. Their depth enables automatic extraction of hierarchical data features, allowing them to handle complex patterns and large datasets effectively. Applied to DNAm data for deconvolution, DNNs use their multi-layered structure to extract intricate features from methylation patterns. Training involves adjusting connection weights based on methylation data and enhancing comprehension of cellular composition in mixed samples. Through comparisons with known reference values, DNNs optimize predicting cell-type proportions. Their capability to learn complex patterns from methylation data makes them valuable for deciphering cellular compositions and estimating cell-type proportions in intricate biological samples.

Hierarchical methods use the algorithms described above in a hierarchical way by performing the deconvolution in different layers. By iteratively estimating the proportions of each component in the mixture, hierarchical deconvolution methods can effectively disentangle complex data into its constituent parts. Two such methods that apply the Houseman’s CP/QP deconvolution algorithm in different layers are ‘Hierarchical Tumor Immune Microenvironment Deconvolution’ (HiTIMED) [76] and ‘Hierarchical Brain Extended Deconvolution’ (HiBED) [77]. In HiTIMED, a six-layers approach (and respective reference sets/libraries) is used to deconvolve and explore the tumour microenvironment of 20 types of carcinomas: in the first layer, a distinction between the tumour fraction from other cell types is made; in the second layer, tumour, angiogenic and immune components are separated; and in the third to sixth layers, specific libraries are used to distinguish between different angiogenic and immune cell subtypes. In the case of HiBED, two layers are applied to distinguish between different brain cells: in the first layer, the proportions for neuronal, glial and endothelial and stromal cells are estimated; the second layer does the same for GABA, GLU, astrocytes, oligodendrocytes, microglia, endothelial cells and stromal cells.

The performance of reference-based methods in estimating cell-type proportions largely depends on the quality, purity and comprehensiveness of the reference dataset. Specifically, deconvolution studies on RNA-sequencing data [15] indicate that these methods perform optimally when the reference set is complete, encompassing all cell types or tissues present in the mixed sample, which is not always feasible. If no reference data or reference data from only part of the cell types are available, unsupervised or semi-supervised methods will offer the favoured deconvolution solution for the problem.

### Unsupervised or reference-free methods

In cases where no reference data are available on the constituting cell types of a mixed sample or when the constituting cell types are not known, reference-free methods can be applied. These techniques provide an alternative approach that allows for more unbiased and flexible estimation of cell or entity proportions and profiles in the context of DNAm deconvolution without relying on pre-existing reference data. They facilitate the exploration of data without preconceived notions, allowing for a broader examination of cellular diversity and the potential revelation of previously undiscovered patterns or relationships within the data. However, a major downside of unsupervised deconvolution tools is that most tools are more computationally intensive, complex and potentially ambiguous as they heavily rely on assumptions. These assumptions encompass various restricting factors such as the mixture samples being composed of a specific number of cell types, the methylation profiles of these samples representing a weighted average of the methylation profiles linked to the underlying cell types and the presence of unobserved or latent variables (methylation profiles) in the process. Some of these tools, such as MethylPurify [78], are only designed towards tumour purity deconvolution and therefore assume that the tissue constitutes of two major components, being healthy and tumour cells.

The lack of a reference set results in unlabelled proportions after deconvolution that might be challenging to interpret.

1) Most of the unsupervised methods use Non-Negative Matrix Factorization (NMF) approaches (RefFreeEWAS [79], RefFreeCellMix [80], EDec [81] and MeDeCom [62]). This is an approach that can decompose a given matrix (the DNAm profile of *m* samples) into two non-negative matrices, one representing the features (the estimated methylation profile of *n* references) and the other representing the coefficients (the proportions for each of the *m* samples of each entity *n*). By finding the best combination of these matrices, NMF can approximate the original data matrix and extract meaningful features. The first reference-free tool developed that was presented by the Houseman’s group in 2014 [79] also falls under this category. The R package referred to as RefFreeEWAS, where RefFreeCellMix is also featured, was developed specifically for conducting epigenome-wide association studies (EWASs). This approach also shares similarities with the technique known as (independent) surrogate variable analysis (SVA and ISVA, respectively) and considers the peripheral blood mononuclear cell (PBMC) samples as whole blood with granulocytes removed (views the composition of whole blood as the sum of the monocytes fraction plus the fraction of the lymphocytes).
2) Other approaches employ Expectation–Maximization (EM) algorithms (MethylPurify and PRISM [78, 82]); these are iterative algorithms used to estimate parameters in statistical models with hidden or unobserved variables (methylation profiles of reference entities). These algorithms alternate between two essential steps: the expectation step, which computes the expected values of the reference profiles given the observed data (methylation profiles of mixed samples) and current parameter estimates, and the maximization step, which updates the parameter estimates based on the computed expected values. Through an iterative process of these two steps, the EM algorithms gradually refine the parameter estimates until convergence is achieved, either when the log-likelihood plateaus or when stability is achieved in the estimated parameters; this convergence can also be reached when a convergence threshold or maximum number of iterations set by the user is satisfied, resulting on outputted estimated proportions.
3) Additionally, a few methods also use the Hidden Markov Model (HMM), like DXM [83], which is a statistical model utilized to analyse data in a sequential manner, where each data point corresponds to an underlying hidden state, and can accurately deconvolve up to three entities. The model iteratively determines the number of entities, starting with a major one and carefully adding more to avoid overfitting. It calculates the optimal prevalence of subpopulations based on observed fractional methylation values, minimizing differences between expected and observed methylation distributions. The Viterbi algorithm within a modified HMM is then used to ascertain the most probable methylation profiles given the known subpopulation count and expected prevalence. The DXM’s HMM assesses the likelihood of individual methylation profiles contributing to observed bisulphite sequencing data. It further expands this assessment to examine the impact of profile combinations on the data, treating the collective methylation sequences of CpGs across all profiles as a sequential arrangement of hidden states. In this framework, transition probabilities signify the probability of a cell type possessing a particular underlying methylation sequence for its CpGs, taking into consideration the influence of CpG site proximity on shared methylation states.

### Hybrid methods

Hybrid deconvolution methods refer to approaches that combine both unsupervised techniques and statistical methods, prior knowledge or supervised techniques to perform deconvolution tasks. These methods aim to leverage the strengths of both approaches to improve the accuracy, robustness and versatility of the deconvolution process, as well as decrease the computational power required for such analysis.

Such methods are referred to as semi-supervised or partially-reference-based deconvolution and include CelFiE [38], EMeth [67], PRMeth [84] and CelFEER [72]. In these approaches, a small set of labelled reference data is used, leveraging the information it provides in conjunction with unsupervised algorithms, such as EM (for CelFiE, EMeth and CelFEER) or iteratively optimized NMF (for PRMeth). The labelled data provide some supervision of the deconvolution process, helping to refine the results and reducing the complexity of the interpretation compared to pure reference-free methods. Other than using a partial reference dataset, the use of known DMRs/DMPs identified by comparing different cell entities or other prior knowledge for data filtering and pre-processing in combination with unsupervised methods, such as in MeDeCom [62], can also be considered a hybrid approach.

A last group of hybrid methods combine reference-based and reference-free algorithms, each trained with different techniques or assumptions. The outputs of the individual models are then combined to generate the result; this approach is also called ensemble learning. This approach benefits from utilizing both unsupervised and supervised models to capture various aspects of the data and improve overall performance. One such example is PRMeth: a package that includes not only a novel partial reference method but also an implementation of both reference-based and reference-free Houseman’s approaches, CP/QP and NMF, respectively. In the first step, the reference-free method (NMF) is used to determine proportions for a known number of entities and afterwards, a Recursive-partitioning Mixture Model (RPMM) [85] is used iteratively in combination with the reference-based method (CP/QP). Another is BayesCCE [86], which applies a Bayesian prior to improve the NMF results.

### Mixed methods

While most of the deconvolution methods fall into one of the categories mentioned above, certain tools fit into all those groups. These methods, depending on the settings, possess the capability to perform deconvolution under various conditions [unsupervised or (partially) supervised]. One tool where such a method is applied is Tsisal [87].

Tsisal utilizes the simplex identification via split augmented Lagrangian (SISAL) algorithm [88], which is primarily designed for unmixing/deconvolution of images. One key step, like for many other tools, is the selection of a list of informative CpG sites, which can be acquired by the usage of TOAST [33], a feature selection method that is integrated in the R package together with Tsisal. The general geometric approach for determining the corners of a shape encounters optimization challenges arising from specific restrictions or limitations. SISAL stands out from typical methods by using more flexible constraints instead of strict positivity rules. This change makes SISAL better at handling errors in data and speeds up its work. The tool first finds important points in a shape and then uses these points to figure out how many different cell types are present. It also helps identify if some parts of the data are connected to specific cell types. These crucial points then act as labels for different cell types, which helps us study them further.

This tool is capable of deconvolving a given sample into its constituent entities not only by using exclusively the methylation profile of that sample (unsupervised deconvolution) but also by offering the flexibility of specifying the number of entities and the reference set as optional parameters, allowing fully or partially supervised deconvolution.

### Tools that identify the main contributing cell type

Tools including BED [89], CancerLocator [90], CancerDetector [91] and the Random Forest model from Capper *et al.* [11] are not deconvolution tools in the strict sense. These methods identify the main contributing/the most abundant cell type in a mixture and the total tumour burden in the mixed sample but do not deconvolve all the contributing cell types in a mixture.

BED is a Bayesian inference–based method developed to estimate fractions of non-healthy cells, e.g. the fraction of cancer cells in a tumour biopsy. This semi-free reference method requires the input of only normal tissue data as reference and provides the tumour purity as the output.

Both CancerLocator and its successor, CancerDetector, are reference-based classifiers that make use of a maximum-likelihood estimation (MLE) algorithm to predict the tumour type and fraction in a sample; this is based on a reference set composed of several tumoral samples combined with healthy samples.

Random Forest models are based on decision trees, that make use of an extensive reference set (train set) to determine the entity present in the mixture samples. If proportions are also indicated for each of the samples contained in the reference set, the model can also estimate the fraction of the predicted entities for the samples under study. The previously mentioned brain tumour classifier [11] uses this strategy.

These tools have in common the fact that they do not estimate/infer the complete constitution of the mixed sample but only report on the most abundant cell entity present in the mixed sample or can distinguish between distinct groups, such as healthy and diseased or multiple brain tumour types. In the remainder of the review, we will focus on deconvolution tools that adhere to the strict definition of deconvolution.

### Selection of the approach

As detailed above, there are several deconvolution tools for DNAm data available nowadays, making it challenging to identify the most appropriate approach for a certain research question. The selection of the tool can be made based on the following parameters (summarized in Table 2):

- Data availability: Is a good-quality dataset available to build a reference set? Does the reference set contain data on all possible cell types contributing to the mixed sample? Based on this information, a supervised, unsupervised or hybrid method should be selected.
- Prior knowledge: In case no reference dataset is available and therefore reference-based deconvolution is impossible, other prior knowledge on the contributing cell types in the mixed sample might be available. Access to information regarding the number of entities or cell-type/entity-specific features such as the DMRs/DMPs allow the usage of hybrid methods instead of fully unsupervised methods, increasing the robustness of the results.
- Data platform: What type of data (e.g. array or sequencing-based data) is available? Certain tools were not developed and cannot (easily) be adapted to array data, such as is the case for CelFiE [38], UXM [65], CelFEER [72] and cfSort [71], that were developed for sequencing-based methylation datasets.
- Available computational power: Unsupervised and hybrid tools demand significant computational resources, including substantial RAM and processing time, to conduct the computational deconvolution of cell-type or entity fractions. However, certain supervised tools that apply iterative procedures also require a large amount of computing power, like hierarchical methods.
- Expertise in parameterization: Many tools depend on correct parametrization (such as threshold selection for beta value significance or selection of algorithm-specific parameters) to produce qualitative results, but there is the potential risk of overfitting the method to the available data. The risk of overfitting arises when the parameters are fine-tuned or adjusted to such an extent that the method becomes overly specialized or tailored to the specific dataset. For example, CelFiE [38] is a tool for which many parameters need to be set.
- Result interpretation: Unsupervised methods, although very flexible, produce unlabelled results that are more complex to interpret. The interpretation requires known methylation markers for the different cell entities that can be used to annotate the different fractions. Tools like FactorViz (an interactive R/Shiny-based tool) [78] facilitate this annotation process by providing interactive visualizations of the results obtained with MeDeCom. This visualization tool allows for investigations into how covariates, like age, sex and tumour stage, affect estimated proportions and Latent Methylation Components (LMCs), associations with technical or phenotypic traits, connections with marker gene expression, survival analysis and functional annotation via Gene Ontology (GO) and Locus Overlap Analysis (LOLA) enrichment analysis. Additionally, users can compare the LMC matrix with reference cell-type profiles for further insights.
- Robustness, sensitivity and performance evaluation: The selection of deconvolution tools involves the evaluation of its performance. Some tools rely on parameters, but not all significantly impact deconvolution outcomes. Robust tools demonstrate consistent and accurate performance across diverse settings, effectively handling unexpected or noisy data and parameter value changes without considerable performance degradation. Certain tools can be especially beneficial in scenarios like low tumour burden in oncological samples, exhibiting higher sensitivity. These tools respond more keenly to input variations, particularly in detecting subtle changes, leading to varied outcomes based on these alterations. Due to this, the research question necessitates careful consideration during tool selection. Moreover, existing benchmarking studies, usually conducted by tool authors, often exhibit favourable conditions for the presented tools in terms of data selection, pre-processing and parameterization, highlighting the critical need for independent and unbiased assessments of tool performance.

**Table 2:** Summary of the parameters to consider when selecting a tool/method. This table works as a guiding point for the selection of the method since the tools benchmarking of the referred parameters and tools fall beyond the scope of this work.

	Reference-based	Reference-free	Hybrid
Reference data required	Yes, all potential entities should be represented in the reference dataset	No	Partially, reference data of some entities may be missing
Prior knowledge and feature selection	Not required but advised to increase accuracy	Not required but will reduce computational run time	Not required but advised to increase accuracy and facilitate predictions of unknown entities
Data platform	Works on both array and sequencing data, however UXM and cfSort only start from sequencing data	Works on both array and sequencing data, but PRISM requires BAM files and feature selection is advised for sequencing data to reduce complexity and run time	Works on both array and sequencing data, however CelFiE and CelFEER only work on raw sequencing data
Available computational power	Low RAM requirements, but some iteration dependent methods require more computational time and power	High RAM requirements and long run times	Medium to high RAM requirements
Parameterization expertise	Dependent on the algorithm, requires benchmarking		
Result interpretation	Easy interpretation since the entities and corresponding proportions are clearly indicated	Difficult interpretation since estimated fraction are not associated with entities	Easy interpretation for the known fractions and difficult for the unknown entities (higher number of unknowns will increase the difficulty of interpretation)
Performance (sensitivity and robustness)	Dependent on the algorithm, requires benchmarking		

## DISCUSSION

The DNAm deconvolution challenge centres on determining the relative proportions of diverse cell types or subpopulations present in a composite sample by analysing the DNAm profiles. When DNAm data are generated on a mixed sample, it captures a composite signal from numerous contributing cell types. Deconvolution hinges upon the diversity of DNAm profiles among discrete cell types. Its objective is to unravel complex mixtures, discerning the unique contributions of each cell type. This process facilitates a finer understanding of DNAm dynamics within particular cellular contexts. This concept finds practical use in various domains, such as oncology, immunology and developmental biology.

In recent times, notable progress has been made in the field of DNAm deconvolution, leading to the creation of diverse tools and methodologies. In this review, we give a comprehensive overview of 25 computational deconvolution methods that have been described between 2012 and 2023, as well as some data-mining steps that can influence the deconvolution results. Indeed, data quality and data pre-processing are fundamental in the analysis process of methylation array or sequencing data and therefore can significantly influence the deconvolution results. Differential methylation analysis for the selection of tissue informative markers is a regularly applied data-mining step that may positively influence the deconvolution results.

Most deconvolution tools available to date are classified as supervised or reference-based methods and use a variety of underlying models including CP, RPC, DNN, LS regressions and SVR methods, the latter two being the most common approaches. A pivotal element in these deconvolution methods is the quality of the reference dataset, which encompasses methylation profiles of the different cell types that compose the mixed sample under study. Such profiles serve as a foundation for accurately deciphering the composition of mixed samples. The quality, purity and completeness of the reference set play a role in obtaining reliable and precise deconvolution results, and although data from multiple platforms can be combined in the reference set, batch correction is essential for the performance of the tools.

On the other hand, unsupervised or reference-free deconvolution methods offer a more unbiased and flexible estimation of cell proportions. These methods prove particularly valuable when reference data are unavailable. Unsupervised methods employ algorithms such as NMF, EM and HMM to extract meaningful features and patterns from methylation profiles to deconvolve the cell proportions. However, unsupervised methods tend to be computationally intensive, complex and rely on certain assumptions. Furthermore, the interpretation of the resulting unlabelled proportions is much more challenging.

To improve the precision and resilience of deconvolution, hybrid approaches have emerged, integrating elements of both unsupervised and supervised techniques. These methods strive to harness the advantages of both approaches, yielding outcomes that are more accurate and adaptable. Semi-supervised or partially based deconvolution methods utilize a small amount of labelled data in conjunction with unsupervised techniques, while hybrid methods combine multiple deconvolution models trained with different techniques or assumptions. By integrating unsupervised and supervised techniques, these hybrid methods yield more precise and reliable deconvolution results while reducing computational requirements.

The evaluation of the performance of deconvolution methods requires ground-truth proportion data of mixed samples. The usage of artificial (*in silico* or *in vitro*) mixtures of known proportions is one of the forms of benchmarking studies to validate the results obtained by the evaluated methods. Metrics such as correlation coefficients (Pearson, Spearman, Lin’s concordance, …) [38, 54, 60, 67–69, 73–76, 86, 87], root mean square error (RMSE) [60, 62, 67, 69, 79], adjusted R-squared [16, 38, 60, 68, 75, 76, 86], accuracy [73, 83] and *P*-value [16, 38, 54, 73–75, 78, 84] can be employed to gauge the accuracy and goodness of fit of the deconvolved proportions.

In the domain of RNA-expression data deconvolution, tools have emerged that utilized single-cell RNA sequencing (scRNA-seq) data instead of bulk RNA-Seq data as a reference. In contrast to bulk sequencing, single-cell analysis explores the cellular diversity and variations between individual cells, resulting in more comprehensive reference sets for deconvolution. Given the rapid technological evolutions during the past few years, we are hopeful that single-cell DNAm profiling will become more feasible soon. Indeed, current technologies developed for single-cell DNAm profiling mostly yield noisy and sparse data. Recently, as an alternative, EPISCORE [73] and scDeconv [92] have emerged as two methods that utilize scRNA-seq data to uncover the composition from bulk DNAm data, known as trans-omics deconvolution.

In conclusion, during the past years, significant advancements have been achieved in the field of DNAm deconvolution, providing a wide range of tools and methodologies to generate a better view of the composition of a complex mixed sample. The integration of diverse data types, the advancement of single-cell analysis techniques and the development of hybrid approaches hold further potential for advancing the accuracy and resolution of deconvolution of bulk DNAm profiles of complex samples.

**Key Points**

- Computational deconvolution of DNAm data from mixed samples estimates the contributing cell-type proportions.
- DNA-methylation deconvolution tools can be classified into supervised, unsupervised and hybrid methods, all having their own strengths and limitations.
- The choice of deconvolution methods depends on factors such as the availability, quality and completeness of reference data; the type of methylation data available and the platform used to obtain it; the sample size and resolution; and the desired level of accuracy and the experimental question to be answered.

## Supplementary Material

Supplementary_File_1_bbae234

Supplementary_File_2_bbae234

## Data Availability

The data supporting the findings of this study are available within the article and its supplementary materials.

## Author Biographies

**Maisa R. Ferro Dos Santos** is a Portuguese PhD candidate at Ghent University and working in the team of Katleen De Preter. She is a bioinformatician focusing on computational deconvolution methods for tumour fraction prediction using epigenomic data.

**Katleen De Preter** is a professor at Ghent University and group leader at VIB-UGent Center for Medical Biotechnology. Her team, Translational Oncogenomics and Bio-Informatics (TOBI), focuses on the development of novel analytical and bioinformatic pipelines for cancer biomarkers.
